# On the Effect of Intra- and Inter-Node Sampling Variability on Operational Modal Parameters in a Digital MEMS-Based Accelerometer Sensor Network for SHM: A Preliminary Numerical Investigation

**DOI:** 10.3390/s25165044

**Published:** 2025-08-14

**Authors:** Matteo Brambilla, Paolo Chiariotti, Alfredo Cigada

**Affiliations:** Politecnico di Milano, Department of Mechanical Engineering, Via Privata Giuseppe La Masa 1, 20156 Milano, Italy; matteo.brambilla@polimi.it (M.B.); alfredo.cigada@polimi.it (A.C.)

**Keywords:** structural health monitoring (SHM), operational modal analysis (OMA), MEMS digital sensor networks, Monte Carlo analysis

## Abstract

Reliable estimation of operational modal parameters is essential in structural health monitoring (SHM), particularly when these parameters serve as damage-sensitive features. Modern distributed monitoring systems, often employing digital MEMS accelerometers, must account for timing uncertainties across sensor networks. Clock irregularities can lead to non-deterministic sampling, introducing uncertainty in the identification of modal parameters. In this paper, the effects of timing variability throughout the network are propagated to the final modal quantities through a Monte-Carlo-based framework. The modal parameters are identified using the covariance-driven stochastic subspace identification (SSI-COV) algorithm. A finite element model of a steel cantilever beam serves as a test case, with timing irregularities modeled probabilistically to simulate variations in sensing node clock stability. The results demonstrate that clock variability at both intra-node and inter-node levels significantly influences mode shape estimation and introduces systematic biases in the identified natural frequencies and damping ratios. The confidence intervals are calculated, showing increased uncertainty with greater timing irregularity. Furthermore, the study examines how clock variability impacts damage detection, offering metrological insights into the limitations of distributed vibration-based SHM systems. Overall, the findings offer guidance for designing and deploying monitoring systems with independently timed nodes, aiming to enhance their reliability and robustness.

## 1. Introduction

### 1.1. Background on Digital Sensor Nodes for Vibration-Based SHM

Structural health monitoring (SHM) [1] encompasses strategies to track the evolution of a system over time, providing timely warnings when potential structural changes are detected (e.g., [2,3]). Raw measurements are first collected through sensors and then processed to extract damage-sensitive features. When structural changes occur, such features are expected to deviate from a healthy baseline state. In this work, the use of damage-sensitive features estimated from acceleration measurements is considered. To this end, accelerometer sensor nodes are deployed along specific degrees of freedom (DOFs) of the monitored system.

In the case of civil structures, operational modal analysis (OMA) [4] is among the most widely used approaches to extract structural parameters. OMA is particularly advantageous for large systems, such as buildings and bridges, where active excitation is challenging, but natural excitations (earth tremors, wind, traffic, and human activity) are stochastic enough, at least within limited bandwidths. Operational modal parameters include, for a specific resonance *j*, the natural frequency $\omega_{j}/2\pi$, the modal damping $\zeta_{j}$, and the mode shape $\psi_{j}$; while the first two are scalar values, the latter is a vector of *S* elements, with *S* representing the number of observed DOFs.

OMA requires the target system to be linear, the natural excitation g(t) stochastic, and the output acceleration data records to be collected synchronously. Although the first two conditions are typically satisfied, the latter is not always guaranteed, especially in scalable and wireless sensor networks (WSN) [5,6,7] based on digital sensor nodes.

**Figure 1:**
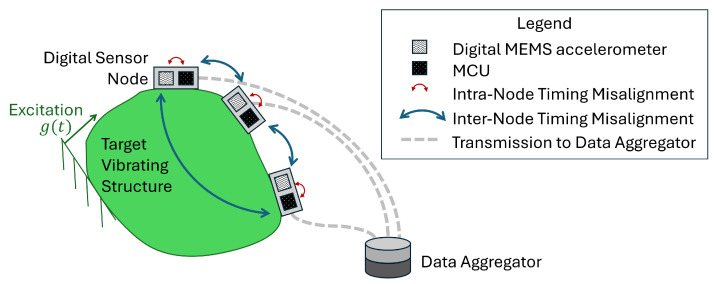
Schematic representation of a digital sensor network for OMA-based SHM.

The schematic of a digital sensor network for OMA-based SHM is shown in Figure 1. Each digital sensor node consists of a digital accelerometer module based on micro-electro-mechanical system (MEMS) technology, paired with a micro-controller unit (MCU) [6,8]. The term *digital* indicates that the accelerometer provides the MCU with already digitized data, as the sensing module integrates analog-to-digital converters (ADCs) and, in some cases, internal sensor processing units (ISPUs). This architecture makes digital sensor nodes a compelling alternative to traditional piezoelectric accelerometers. Furthermore, the availability of low-cost spare parts enables large-scale deployments with hardware redundancy, while MCU and ISPU can be used for onboard data processing on the *edge* of the network, within the node [9,10].

Despite these advantages, MEMS-based accelerometers are generally considered to be less performant from a metrological standpoint compared to high-standard piezoelectric accelerometers. They typically exhibit a higher noise floor and greater sensitivity to temperature fluctuations, aging, frequency dependence, and amplitude variations. In the case of OMA-based applications, the primary concern for effective deployment of digital sensors lies in the reliability of the sampling rate within individual sensor nodes and the ability to achieve simultaneous sampling between nodes in the sensor network (Figure 1). When a common time reference cannot be shared among sensors belonging to the same network, the physical phase relationship among the sensorized DOFs is lost. Consequently, operational modal parameters can deviate from baseline even under undamaged conditions, leading to false alarms or masking structural alterations, resulting in missed detections.

This paper, therefore, specifically focuses on the issue of lack of synchronization and does not consider the combined effects of other sources of uncertainty. Time misalignment occurs at two distinct levels within the sensor network (Figure 1). At the intra-node level, i.e., within a single digital node, the presence of multiple clock sources (e.g., in the ADC and MCU) or the instability of non-quartz-based clock sources results in data records sampled at non-constant time intervals. At the inter-node level, the lack of a master time reference among the sensor nodes leads to non-simultaneous sampling among DOFs. A first attempt to experimentally quantify sampling variability was conducted by D’Emilia et al. [6], in which a set of 25 MEMS-based sensors with digital interfaces—sourced from the same production batch—were tested at selected sine-dwell frequencies. The results revealed significant variability in the actual sampling rates among the sensors, as well as noticeable deviations from the nominal rate specified by the manufacturer. This fundamental issue must be addressed both during dynamic calibration [11] and under operational conditions, such as in SHM applications. The present work focuses on the latter.

Focusing on linear time-invariant (LTI) structures under stochastic ambient excitation, several approaches have been proposed to address the issue of lack of synchronization. In the context of wireless sensor networks (WSNs), Lei et al. [12] examined time delays caused by different triggering times, radio transmission interference, and internal sensor clock errors, highlighting that non-simultaneous sampling primarily affects mode shape identification rather than natural frequencies and damping ratios. They proposed two synchronization algorithms to improve damage detection for both known and unknown input conditions. Alternatively, assuming that the first mode eigenvector is real (i.e., zero-phase), Bernal [13] derived a characteristic phase metric to correct for the misalignment error without directly estimating time delays. Zhou et al. [14] addressed the problem of realigning sensors with constant sampling rates, where the lack of synchronization is primarily caused by initial clock offsets. Given a reference signal, the time delay of a second signal is estimated in the time domain based on its phase shift. In the case of a mode where the phase angle between the two signals is ideally zero, the phase shift is retrieved from the analysis of cross-power spectral densities. Dragos et al. [9] proposed an onboard real-time phase correction method for WSNs, where synchronization is based on phase angle relationships at modal peaks in the Fourier amplitude spectra of acceleration response signals. Later, in [15], they demonstrated that when real-time synchronization is infeasible, post-processing corrections can achieve time synchronization using cross-spectral density phase analysis. Huang et al. [10] introduced a decentralized framework for OMA-based SHM that eliminates the need for inter-sensor synchronization by relying solely on local measurements. Instead of transmitting raw measurement data, only the model parameters and their covariance matrices are shared. Shajihan et al. [16] focused on clock oscillation issues within the internal ADCs of digital accelerometers. Using the Flooding Time Synchronization Protocol and an adaptive delay estimation algorithm, they achieved a clock synchronization error of 15 μs.

Despite the many alternatives proposed in the literature, the lack of synchronization cannot be completely eliminated. Moreover, the varying assumptions and boundary conditions of each method make it challenging to select the most appropriate correction strategy. However, even under (contained) uncertain sampling conditions, a digital sensor network can still be effectively used for SHM. For instance, Kullaa [17] demonstrated that strict synchronization is not always necessary if auto-covariance functions (ACFs) are used as damage-sensitive features. This aligns with the stability of the scalar operational parameters observed in [12] and later in [18], where lack of synchronization was modeled as a limited drift in time records to simulate sensor clock misalignment when a global clock reference is missing. Their findings indicate that natural frequencies remain reasonably unaffected, while damping values tend to be significantly overestimated. Mode shapes are the modal quantities most affected by lack of synchronization and require corrections for reliable use in SHM applications.

Many applications may benefit more from quantifying the uncertainty in modal parameters under uncontrolled timing conditions rather than focusing solely on restoring synchronism in data records. The confidence interval metric for modal parameters estimated under stochastic conditions has been well established for aligned and properly sampled data in [19,20,21].

Moreover, recent works [22,23] have emphasized that uncertainty quantification is not optional in SHM; it is central to ensuring safety and reliability when data volatility is high. Uncertainty, measured through modelling errors and confidence intervals, increases significantly under extreme wind and traffic conditions. Quantitative uncertainty analysis can identify environmental drivers of measurement error, thereby supporting more reliable early warning and maintenance decisions.

### 1.2. Purpose of the Work and Preliminary Assumptions

Building upon a few prior seminal works on estimating operational modal parameters under timing misalignment conditions (e.g., [18]), this paper proposes a methodological approach to quantify the effect of sampling variability on operational modal parameters estimated from accelerations collected through a digital sensor networks affected by sampling variability.

This is motivated by the fact that, although digital sensor network solutions offer clear advantages in terms of cost, maintainability, and data handling, they require careful consideration by SHM designers.

This paper contributes to the growing body of research on the metrological performance of digital sensor networks, applied to the well-established context of vibration-based SHM of civil structures using digital accelerometers. Sampling variability is modeled by accounting for the firmware architecture within the digital node, and the performance of the OMA-based SHM application is assessed in terms of reliability of damage-sensitive features under different structural conditions.

The overall uncertainty depends on the specific SHM application and is influenced by several interdependent variables. The results presented refer to a specific test case and are not intended to be universally generalized. Instead, they support an informed use of the proposed framework.

Under synchronous conditions, various interdependent factors are known to influence the estimated OMA parameters and their reliability. These interactions can overlap, obscure, or bias the impact of sampling variability, which is the primary focus of this study. The early-stage framework is developed by fixing, simplifying, or neglecting the following sources of variability:

**Modal complexity of the target structure.** Modal identification is more challenging in structures with closely spaced modes (e.g., symmetric systems), compliant boundary conditions, or high damping. For this preliminary investigation, a cantilever beam with linear structural damping is selected as an academic benchmark.
**Environmental and Operational Variables (EOVs).** Environmental factors—particularly temperature—are known to affect the reliability of OMA-based damage detection, as they influence the modal parameters of undamaged structures [24]. Although robust compensation techniques exist (e.g., [25]), they are typically validated using high-quality, synchronous sensor networks. Other EOVs, such as humidity and loading conditions, may also bias both the structure and low-cost sensor components. The interaction between EOVs and sampling variability represents a complex scenario and is beyond the scope of this work. In this study, EOVs are assumed constant, and the same stochastic excitation is applied across all cases.
**Sensor placement** [26]. Avoiding spatial aliasing is critical. The number and placement of sensors are selected to ensure accurate modal identification under synchronous conditions without excessive computational cost. An equally spaced array of digital sensors is deployed along the cantilever beam.
**Signal-to-noise ratio (SNR).** SNR depends on the specific performance of the selected sensors and the dynamic behavior of the target structure [27]. In OMA, it directly relates to operating conditions, as accelerometers must be sensitive to the expected vibration range. Prior works (e.g., [28,29]) show that SNR effects in modal identification can be mitigated through algorithmic adjustments, such as increasing the model order. In this study, low SNR is not treated as a source of variability.

These considerations justify the use of a numerical model (see Section 2.1) for this investigation.

The choice of the modal parameter extraction algorithm directly affects the accuracy of the identification. State-of-the-art algorithms assume data to be collected with constant sampling intervals. Several methods are available for extracting OMA parameters [30,31]. Among them, Frequency Domain Decomposition (FDD) is known for robustness in low SNR conditions, while covariance-driven stochastic subspace identification (SSI-COV) can outperform FDD and data-driven SSI in terms of speed if properly tuned [32].

Comparing the robustness of different algorithms to sampling variability is beyond the scope of this work. Instead, this study focuses on evaluating the performance of digital sensor networks within a specific SHM application. Notably, previous works based on different identification strategies, such as [12] (using ARMAV models) and [13] (using SSI with corrective actions), arrived at the same conclusions: synchronization errors significantly affect mode shape estimation but have limited impact on frequency and damping estimates.

In this work, the SSI-COV algorithm is adopted to study the identification of operational modal parameters under sampling variability. Since SSI-COV involves complex parameter estimation, traditional analytical uncertainty propagation is difficult. Monte Carlo methods are well suited for uncertainty propagation in non-linear pipelines, like this one that involves a modal identification step. By repeatedly running the identification algorithm on perturbed input data, the Monte Carlo framework enables empirical estimation of the probability distributions of the modal parameters, providing valuable metrics such as standard deviation and confidence intervals for SHM and reliability assessment.

In addition, SSI-COV integrates seamlessly into a Monte Carlo framework. It is computationally efficient, robust, and well suited for automation, making it ideal for reproducible modal parameter extraction [20,33,34,35].

### 1.3. Structure of the Paper

The paper is organized as follows: In Section 2.1, the target SHM application is introduced. Section 2.2 provides a brief overview of the stochastic subspace identification algorithm along with the parameter settings used for automatic estimation of operational modal parameters. Section 3 describes the statistical modeling of time misalignment affecting both individual nodes and the overall sensor network. This step is particularly important, as the level of insight into the firmware architecture significantly influences the input uncertainty in the Monte Carlo framework—specifically, the non-deterministic sampling variability.

These components are integrated into the Monte Carlo simulation framework in Section 4 to generate datasets of operational modal parameters. The results and associated confidence intervals are analyzed in Section 5, where the impact of non-deterministic sampling variability on SHM performance is evaluated in two steps. First, the framework estimates the uncertainty in the undamaged configuration, where the beam is assumed to be healthy. Then, the same process is applied to the damaged configuration. This allows the evaluation of damage sensitivity using the digital sensor network by assessing the ability of damage-sensitive features to deviate from the healthy baseline. Finally, the main conclusions are presented in Section 6.

## 2. Materials and Methods

### 2.1. Target Vibrating System

**Figure 2:**
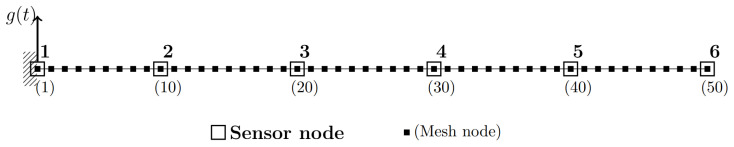
Numerical model of the cantilever beam. Input excitation, g(t), is applied at the grounded mesh node 1. Beam elements are numbered from 1 to 49 from left to right.

The structure to be used as a data generator is a 5 m long cantilever beam with a rectangular cross-section of 0.012 m × 0.006 m. The beam was modeled as a two-dimensional linear structure in Abaqus [36], with transverse vibrations along its 0.006 m thickness considered as the DOFs of interest. The beam is made of steel, with a mass density of 7800 kg/m^3^, a Young’s modulus *E* of 210 GPa, and a Poisson’s coefficient of 0.3. In Figure 2, the finite element (FE) mesh is shown, along with the locations of six virtual accelerometers positioned to measure transverse vibrations relative to the beam axis. The mesh consists of 49 two-dimensional beam elements with linear interpolation (labeled as B12 in the Abaqus nomenclature), with a total of 50 nodes, where node 1 represents the clamped edge and node 50 corresponds to the free edge. The damping matrix D is modeled as proportional to mass and stiffness matrices M and K, such that:

(1) D=γM+βK

with γ and β equal to 0.05 $s^{-1}$ and $3.5\times 10^{-5}$ s respectively. It is important to note that the sensors are not explicitly modeled in the FE model; however, the time records of the total acceleration along those DOFs are captured. The mass of the sensors is also considered null with respect to the local mass of the beam at the virtual sensor locations.

The eigenvalue analysis on the FE model is used to compute the natural frequencies $\omega_{j}$ and mode shapes $\phi_{j}$ of the undamped system:

(2) $$M\ddot{x}\left(t\right)+Kx\left(t\right)\;=\;0\;\to \;\left(K-\omega^{2}M\right)Xe^{i\omega t}\;=\;0$$

**Figure 3:**
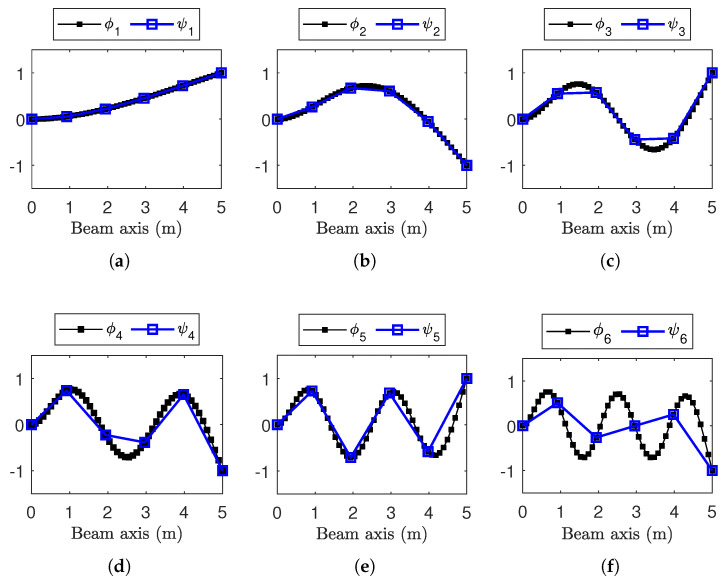
Mode shapes from the eigenvalue analysis. (**a**–**f**) Resonances from j=1 to j=6. Consistently with Figure 2, $\phi_{j}$ indicates results for the entire mesh as filled squares (■); $\psi_{j}$ highlights with squares (□) those in sensor node location.

Here, $x\left(t\right)\;=\;Xe^{i\omega t}$ is the trial solution to Equation (2), encompassing all the DOFs of the FE model. At resonance *j*, the corresponding mode shape $\phi_{j}$ is obtained by substituting $\omega_{j}$ back into Equation (2). The results of the eigenvalue analysis for the first six modes are reported in terms of the natural frequency and normalized mode shapes in Table 1 and Figure 3, respectively.

To simulate a practical SHM application, where only a subset of the structure DOFs is equipped with sensors, the mode shapes $\phi_{j}$ are henceforth denoted as $\psi_{j}$, representing $\phi_{j}$ only at the sensor locations (i.e., $\psi_{j}\subseteq \phi_{j}$). This implicitly restricts the analysis up to the fifth resonance (j=5), since the SHM system depicted in Figure 2 consists of only S=6 sensor nodes (sensitive to the DOF transversal to the beam axis), and spatial aliasing occurs from j=6 (Figure 3f) onward.

The modal progression presented in Table 1 demonstrates well-separated resonances in the frequency domain. This enables the selection of an appropriate frequency bandwidth for the random excitation signal g(t), used to generate synchronous time-domain data records $y_{s}$, where the subscript s=1,…,S denotes the sensor node.

Moving to damage scenarios, structural alterations are modeled as a homogeneous reduction in stiffness at specific elements of the FE mesh. The Young’s modulus of the damaged elements, $E_{d}$, is obtained from one of the baselines, *E*, through a stiffness reduction coefficient δ, as shown in Equation (3).

(3) $$E_{d}\;=\;\left(1-\delta \right)E$$

Referring to Figure 2, three damage locations are considered separately.

- d1: elements from 9 to 11 (corresponding to sensor s=2).
- d2: elements from 4 to 6 (between sensors s=1 and s=2).
- d3: elements from 1 to 49 (the entire beam suffers from a stiffness reduction).

Localized damages, d1 and d2, involve three consecutive elements of the FE mesh, while distributed damage, d3, corresponds to the same reduction in stiffness in all elements of the mesh. Different severity levels, labeled for increasing values of δ, are reported in Table 2. The value of δ is intended differently for localized and distributed damage: in the first case, δ indicates the reduction in stiffness in the central element of the three elements that contribute to the damaged zone, while the lateral elements are to be considered for a reduction equal to δ/2; in the second case, the reduction in stiffness δ applies to all elements of the beam FE mesh.

### 2.2. Operational Modal Parameter Estimation via SSI-COV

Given a set of output-only (nominally simultaneously sampled) data records $y_{s}$, collected at s=1,…,S sensorized DOFs of a structure, the operational modal parameters are obtained by estimating a discrete stochastic state-space model under stochastic excitation:

(4) $$\begin{array}{cc} x[k+1] & =Ax[k]+w[k] \end{array}$$

(5) $$\begin{array}{cc} y[k] & =Cx[k]+v[k] \end{array}$$

At the *k*-th discrete time, x[k] is the state vector, and y[k] is the output vector collecting the data from all $y_{s}$ sensors; A and C are the state and output matrices, respectively; w[k] accounts for disturbances and model inaccuracies, and v[k] represents measurement noise. For a complete theoretical background, the reader is referred to [30,33]. Matrices A and C are estimated from the output responses using the covariance matrices computed from progressively shifted time segments between the output channels $y_{s}$, up to a maximum lag defined by the parameter τ (in seconds).

Once A and C are obtained, eigenvalue analysis on state matrix A yields the operational modal parameters $\omega_{j}$, $\zeta_{j}$ and $\psi_{j}$.

To identify stable poles (i.e., of physical significance rather than numerical), the algorithm is run across model orders *n* ranging from a user-defined minimum $n_{\min}$ to a maximum $n_{\max}$. A stability check is performed using thresholds on relative changes in natural frequency ($\epsilon_{\omega /2\pi }$), damping ratio ($\epsilon_{\zeta }$), and mode shape similarity, with the latter being quantified in terms of the modal assurance criterion (MAC) [37], with a threshold $\epsilon_{\mathrm{MAC}}$ to assess the similarity between modes *q* and *r*.

Once stable poles are selected, a clustering algorithm groups similar poles. The clustering tolerance $\epsilon_{\mathrm{cluster}}$, expressed in Hz, defines the total allowable range for each cluster, meaning the radius spans $\pm \epsilon_{\mathrm{cluster}}/2$.

The eigenvalue analysis of the undamaged condition is used to tune the SSI-COV algorithm. The setup parameters for SSI-COV algorithm [34] include the time lag τ, the stability thresholds $\epsilon_{\omega /2\pi }$, $\epsilon_{\zeta }$, $\epsilon_{\mathrm{MAC}}$, and the cluster range $\epsilon_{\mathrm{cluster}}$. The selected values, reported in Table 3, remain fixed for all Monte Carlo simulations. In addition, the nominal sampling interval $dt=1/f_{S}$ is required as input, although SSI-COV is unaware of the actual fluctuations in sampling affecting the records. Therefore, the sampling rate $f_{S}$ is assumed by SSI-COV as constant.

As the parameter τ defines the maximum time lag for computing cross-covariance, larger values allow impulse response estimation to capture lower-frequency dynamics. However, this comes at the cost of increased computational time [33]. In this study, τ=2 s is selected to allow reliable estimation above 0.5 Hz. Consequently, and according to Table 1, the first resonance (j=1) falls below this limit and is excluded from the analysis. This reflects a trade-off between frequency resolution and computational efficiency, which is especially important for simulating large datasets in Monte Carlo simulations.

Stability thresholds $\epsilon_{\omega /2\pi }$, $\epsilon_{\zeta }$, and $\epsilon_{\mathrm{MAC}}$, as well as the minimum and maximum model orders $n_{\min}$ and $n_{\max}$, have been tuned to ensure consistency with the reference values obtained from the eigenvalue analysis (see Table 1 and Figure 3).

Regarding $\epsilon_{\mathrm{cluster}}$, while a tighter clustering range increases sensitivity to structural alterations, the main objective of this study is to evaluate the performance of SSI-COV when applied to asynchronous records. A stricter clustering criterion might improve sensitivity to structural changes but may also exclude poles that are valid yet slightly shifted due to uncertain sampling. For this reason, a value of $\epsilon_{\mathrm{cluster}}\;=\;4$ Hz (i.e., ±2 Hz) is considered appropriate, as it aligns with the frequency separation of the resonances shown in Table 1.

## 3. Non-Deterministic Sampling Variability in Digital-Sensor-Node Networks

Although SSI-COV is assumed to be used with synchronous signals $y_{s}$ recorded on a target vibrating structure, the aim of this study is to include the effect of an uncertain time-based alignment between the sensing nodes on operational modal parameters. The present section introduces a time-based shifting model that may typically characterize the firmware architecture of a digital MEMS sensor network in the presence of multiple clock signals not sharing a common master time basis nor implementing any correction strategy from the literature.

**Figure 4:**
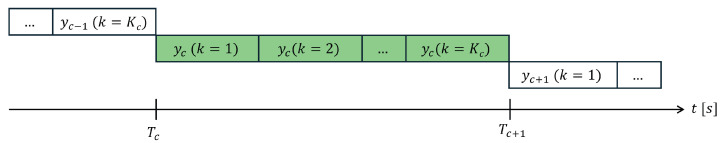
Schematic representation of the circular FIFO queue of the *s*-th digital accelerometer. $K_{c}$ samples complete one chunk (*c*). One timestamp $T_{c}$ is assigned to each chunk in the MCU.

Within the *s*-th digital sensing node, the two main players are the digital accelerometer module, which is devoted to continuously sensing vibrations, and the MCU, which typically manages data aggregation (from data buffer streamed in real-time), communication, and logging. Each digital accelerometer embeds its own clock and temporarily stores digital samples in a first-input-first-output (FIFO) queue. To prevent data loss, the MCU must download the FIFO buffer before it reaches saturation. As shown in Figure 4, once a predefined number of samples per chunk, $k=1,2,\ldots ,K_{c}$ is collected, the *c*-th data chunk for the *s*-th sensing node, $y_{c,s}$, is retrieved.

(6) $$y_{c,s}\;=\;\left[\ldots ,y_{c,s}\left[k\right],\ldots \right]$$

At the beginning of each chunk $y_{c,s}$, the MCU assigns a time reference in the form of a time stamp $T_{c}$, and subsequently transmits it to the central server of the network. Meanwhile, the accelerometer module continues to acquire new data samples, storing them in the FIFO circular buffer in preparation for the next chunk, c+1. Under aligned time stamp conditions, the nominal output data rate for the *s*-th sensor ($ODR_{nl,s}$), that is, the rate at which a buffer of samples is made available to an external actor such as the MCU, is recovered from the timestamps of two consecutive chunks (Equation (7)).

(7) $$ODR_{nl,s}\;=\;\frac{K_{c}}{T_{c,s}-T_{c-1,s}}$$

The output data rate (ODR) is usually considered to be time invariant. If this condition holds, the ODR is approximately equal to the sampling frequency $f_{S}$ of the ADC, even though this simplification is not formally correct. In practice, the absence of shared clock signals within a node and between nodes of the sensor network suggests that the actual ODR may oscillate around $ODR_{nl,s}$ at the individual node level (intra-node variability) and at the network level (inter-node variability).

This variability may arise from factors such as jitter, temperature fluctuations, and hardware faults, making the development of a comprehensive deterministic model overly complex. Given these challenges, a more intuitive and tractable approach is to address the problem from a probabilistic perspective. Assuming that systematic clock drift errors are reasonably compensated by state-of-the-art resynchronization strategies—such as those based on network time protocol (NTP) [38] or global positioning system (GPS)—the non-deterministic component of ODR fluctuations remains. These fluctuations, as well as their impact on the estimation of operational modal parameters, constitute the primary focus of this study.

In [39], the experimental evaluation of the ODR in a digital sensor network shows that it follows a statistical distribution that can be approximated by a Gaussian. In the application case considered in this work, the ODR is then modeled as a random variable that varies both across chunks and between sensor nodes and is assumed to follow a Gaussian distribution, as described in Equation (8).

(8) $$N\left(x|\mu_{x},\sigma_{x}^{2}\right)\;=\;\frac{1}{{\left(2\pi \sigma_{x}^{2}\right)}^{1/2}}\exp\left\{-\frac{1}{2\sigma_{x}^{2}}{\left(x-\mu_{x}\right)}^{2}\right\}$$

In Equation (8), the random variable *x* represents the ODR value for a given chunk in a given node, which is no longer fixed to the constant nominal value $ODR_{nl,s}$. The parameters $\mu_{x}$ and $\sigma_{x}^{2}$ denote the mean and variance of the Gaussian distribution, respectively.

Assuming that systematic clock drifts are effectively compensated, it is reasonable to consider the nominal synchronous ODR, addressed as $ODR_{nl,s}$ for the *s*-th sensing node, to correspond to the mean of the Gaussian distribution (Equation (9)).

(9) $$\mu_{x}=ODR_{nl,s}$$

The non-deterministic sampling variability that affects the digital nodes is quantified using the coefficient of variation of the stochastic ODR variable *x*, denoted as $\alpha_{x}$ in Equation (10).

(10) $$\alpha_{x}\;=\;\frac{\sigma_{x}}{\mu_{x}}$$

In Equation (10), $\alpha_{x}=0$ corresponds to nominally synchronous sampled data conditions, while $\alpha_{x}>0$ quantifies the sampling variability. Larger values of $\alpha_{x}$ correspond to increased uncertainty due to non-deterministic sampling (i.e., strong misalignments in the time bases) in the recorded data.

In the *s*-th sensor (i.e., at intra-node level), the time-variant sampling is statistically modeled in Equation (11) by combining the assumptions outlined in Equations (8)–(10).

(11) $$x_{c,s}\in N\left(x|ODR_{nl},\alpha_{x}^{2}ODR_{nl}^{2}\right)$$

Therefore, if considering the *c*-th data chunk of the *s*-th sensing node, the $K_{c}$ samples are acquired at a constant ODR $x_{c,s}$, meaning that consecutive samples are spaced by a time interval:

(12) $$dt_{c,s}=\frac{1}{x_{c,s}}$$

where $x_{c,s}$ is drawn from the Gaussian distribution of Equation (11).

A final remark concerns the number of samples per chunk, $K_{c}$. The assumption of a constant $K_{c}$ during acquisition is justified by the fact that the firmware architecture is based on interrupt requests, meaning that data chunk transmission is triggered once the FIFO buffer is filled. As a result, a more realistic failure scenario would involve the complete loss of a data chunk, rather than fluctuations in its size. This falls under the broader category of sensor faults [7], and represents an additional source of variability.

In this work, a fixed-duration recording of T=240s is considered. Data loss events, such as missing chunks, are more likely to occur in long-term continuous SHM monitoring. For this reason, $K_{c}$ is not treated as a source of uncertainty in the present case study.

## 4. Monte Carlo Framework for Estimating Modal Parameter Uncertainty Under Non-Synchronous Sampling

The use of digital sensor networks in OMA-based SHM applications is known to be affected by non-deterministic sampling, which occurs at both the intra-node and the inter-node levels. This phenomenon can bias the estimation of operational modal parameters. However, in order to use these parameters as reliable damage-sensitive features, their variability should ideally be attributed only to structural changes. For this reason, the uncertainty introduced by non-deterministic sampling must be quantified to understand the limitations of the sensor network deployed on the monitored structure.

Although the behavior of operational modal parameters in the presence of timing-uncertain conditions has been documented in a few seminal studies (e.g., [12,18]), there is still a lack of a comprehensive methodology capable of systematically accounting for the sources of clock misalignment on the final modal parameters. These sources include the hardware and firmware architecture of the digital sensor nodes and the configuration of the modal identification algorithm applied to a specific vibrating structure.

**Figure 5:**
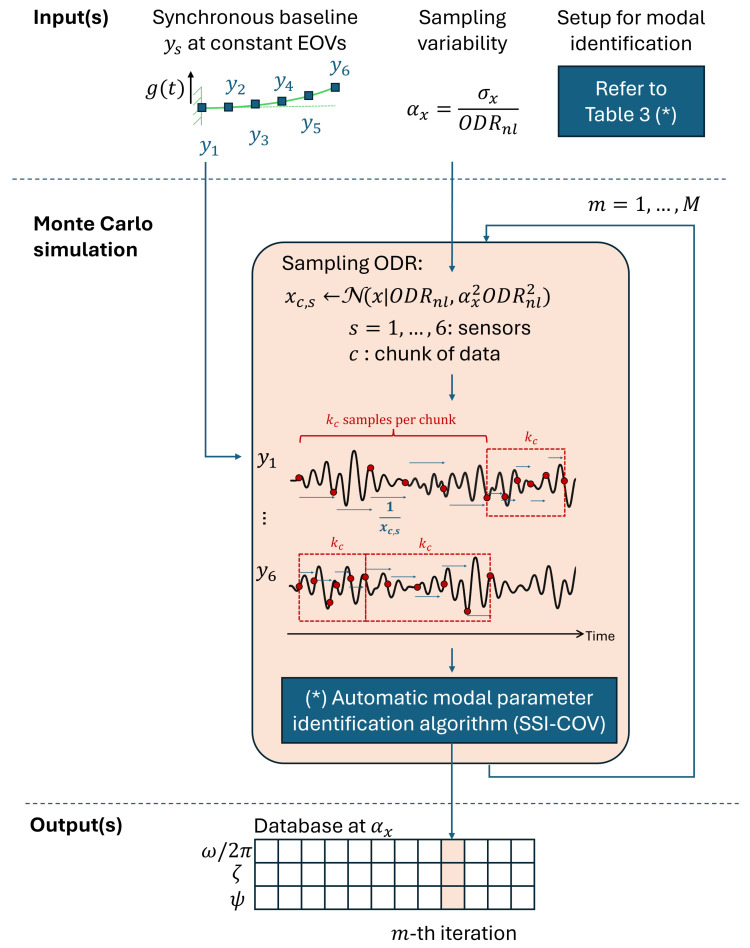
Schematic flowchart of the generation of a dataset of *M* iterations at a given sampling variability $\alpha_{x}$.

To address this gap, a methodology is proposed to quantify the uncertainty in $\left\{\omega_{j}/2\pi ,\;\zeta_{j},\;\psi_{j}\right\}$ due to non-deterministic sampling variability, using a Monte Carlo approach. The underlying logic of the method is illustrated schematically in Figure 5.

In this paper, the numerical model of the vibrating system (Figure 2) plays a dual role. It is used first as a data generator, producing synchronous acceleration records $y_{s}$ for s=1,…,S virtual sensor nodes. Second, it serves as a reference model in which EOVs and algorithmic input parameters (Table 2) remain fixed so that the effect of non-deterministic sampling variability on the operational modal parameters can be isolated and assessed. Although a simple FE model is used in this study, the use of more complex models is also feasible at a reasonable computational cost. For example, partial models (e.g., [2]) can be employed to model just the damaged part.

Generating a dataset for a specific level of non-deterministic sampling variability, $\alpha_{x}$, requires a preliminary step: the generation of synchronous data records.

The SSI-COV algorithm is configured to process acceleration records $y_{s}$, with a total duration of *T* seconds, under a stochastic excitation g(t), such as white-noise ground acceleration up to 150 Hz. Under nominally synchronous conditions, the data record $y_{s}$, corresponding to the DOF associated with the *s*-th sensor on the numerical structure, is sampled at the nominal output data rate $ODR_{nl,s}$. This involves sequentially appending data chunks $y_{c,s}$ (see Equation (6)) for c=1,…,C, up to the total considered time *T*:

(13) $$y_{s}\;=\;\left[y_{1,s},\;y_{2,s},\;\ldots ,\;y_{C,s}\right]$$

As a result, the numerical model provides a reference dataset matrix Y, defined in Equation (14), where each row corresponds to the acceleration data measured at the DOF associated with the *s*-th sensor, sampled at $ODR_{nl,s}$:

(14) $$Y=\left[\begin{array}{cccc} y_{1}\left[1\right] & y_{1}\left[k\right] & \ldots & y_{1}\left[ODR_{nl,1}\times T\right]\\ y_{2}\left[1\right] & y_{2}\left[k\right] & \ldots & y_{2}\left[ODR_{nl,2}\times T\right]\\ \vdots & \vdots & \ddots & \vdots \\ y_{S}\left[1\right] & y_{S}\left[k\right] & \ldots & y_{S}\left[ODR_{nl,S}\times T\right] \end{array}\right]$$

In each structural state (undamaged or damaged: d1, d2, or d3), the reference dataset $Y^{\mathrm{state}}$ is generated under nominal sampling conditions. For clarity, all sensor nodes are hereafter assumed to operate at the same nominal output data rate $ODR_{nl,s}$ and with the same number of samples per chunk $K_{c}=100$, without loss of generality. Accordingly, the subscript *s* may be omitted when referring to $ODR_{nl,s}$.

At this stage, the non-deterministic sampling variability is introduced through the parameter $\alpha_{x}$ (Equation (10)). A single Monte Carlo iteration is illustrated in Algorithm 1 and consists of the following steps. At the intra-node level, a misaligned time basis ${\hat{t}}_{s}\;=\;\left(\ldots ,{\hat{t}}_{s}^{k},\ldots \right)$ is generated for the *s*-th sensor from the distribution defined in Equation (8). The corresponding asynchronous data record ${\hat{y}}_{s}\;=\;\left(\ldots ,{\hat{y}}_{s}\left[{\hat{t}}_{s}^{k}\right],\ldots \right)$ is then derived by linearly interpolating the synchronous signal $y_{s}\;=\;\left(\ldots ,y_{s}\left[t^{i}\right],\ldots \right)$. The interpolation is performed as addressed in Equation (15).

(15) $${\hat{y}}_{s}\left[{\hat{t}}_{s}^{k}\right]\;=\;y_{s}\left[t^{i}\right]+\frac{y_{s}\left[t^{i+1}\right]-y_{s}\left[t^{i}\right]}{t^{i+1}-t^{i}}\cdot \left({\hat{t}}_{s}^{k}-t^{i}\right),\;t^{i}\leq {\hat{t}}_{s}^{k}<t^{i+1}$$

Here, $t^{i}$ and $t^{i+1}$ represent consecutive timestamps from the synchronous time basis t. Note that subscript *s* is intentionally omitted to highlight the simultaneous sampling among sensors in the ideal scenario provided by the numerical model. Inter-node asynchronism arises because each sensor node generates its asynchronous time base independently, in the absence of a shared master clock. The final output of this step is the non-synchronous matrix $\hat{Y}$, organized to match the input format expected by the SSI-COV algorithm, which is configured according to the setup parameters given in Table 3.

Finally, SSI-COV intentionally assumes $\hat{Y}$ to be sampled at nominal ODR ($ODR_{nl}$), forcing the non-deterministic sampling to manifest itself on the operational modal parameters. For each Monte Carlo iteration, SSI-COV provides a triplet composed of the natural frequency $\omega_{j}/2\pi$, the damping ratio $\zeta_{j}$, and the mode shape vector $\psi_{j}$ for each identified resonance.

## 5. Results

The confidence intervals [19,21] for operational modal parameters are defined as ranges centered on the sample mean $\hat{\mu }$ and extending one sample standard deviation $\hat{\sigma }$ in each direction.

Equations (16)–(18) define the confidence intervals Δ for the *j*-th natural frequency, modal damping ratio, and mode shape, respectively, at a given level of non-deterministic sampling variability $\alpha_{x}$:

(16) $$\begin{array}{cc} \Delta \omega_{j}/2\pi \left(\alpha_{x}\right) & ={\hat{\mu }}_{\omega_{j}/2\pi }\left(\alpha_{x}\right)\pm {\hat{\sigma }}_{\omega_{j}/2\pi }\left(\alpha_{x}\right) \end{array}$$

(17) $$\begin{array}{cc} \Delta \zeta_{j}\left(\alpha_{x}\right) & ={\hat{\mu }}_{\zeta_{j}}\left(\alpha_{x}\right)\pm {\hat{\sigma }}_{\zeta_{j}}\left(\alpha_{x}\right) \end{array}$$

(18) $$\begin{array}{cc} \Delta \psi_{j}\left(\alpha_{x}\right) & ={\hat{\mu }}_{\psi_{j}}\left(\alpha_{x}\right)\pm {\hat{\sigma }}_{\psi_{j}}\left(\alpha_{x}\right) \end{array}$$

Note that the analysis is limited to resonances from j=2 to j=5, in accordance with Table 1, as resonance j=1 is excluded due to the selected value of τ in the SSI-COV configuration.

**Figure 6:**
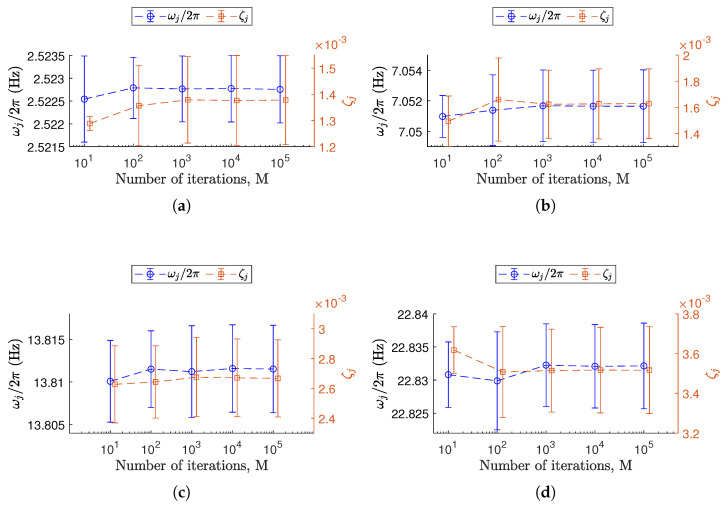
Convergence behavior of Monte Carlo simulations for the representative case with a non-deterministic sampling variability level of $\alpha_{x}\;=\;1.0\%$. (**a**–**d**) Each plot corresponds to a specific *j*-th resonance among those considered, from j=2 to j=5. The confidence intervals for $\omega_{j}/2\pi$ (Equation (16)) refer to the left vertical axis and are shown as error bars centered on circles (∘). The confidence intervals for $\zeta_{j}$ (Equation (17)) refer to the right vertical axis and are reported as error bars centered on squares (□).

For each level of non-deterministic sampling variability $\alpha_{x}$, a total of *M* Monte Carlo iterations yields a dataset containing *M* realizations of the triplet $\left(\omega_{j}/2\pi ,\zeta_{j},\psi_{j}\right)$. The confidence intervals at each $\alpha_{x}$ are then estimated from the corresponding dataset. As a result, the confidence intervals are parameterized over $\alpha_{x}$. The high computational cost required to generate a single database (i.e., for a single level of $\alpha_{x}$) poses significant limitations in reaching a large number ($M=10^{5}$) of iterations. Without using high-performance computing (HPC) resources, it is essential to balance accuracy and computational efficiency. As a practical solution, a preliminary convergence study on a representative case ($\alpha_{x}=1\%$) using $M=10^{5}$ iterations has been observed from a convergence analysis. Since previous works [12,13] suggested that natural frequencies and damping ratios are generally more robust to sampling variability, convergence analysis on those parameters is reported in Figure 6. Convergence to low variability in terms of mean and standard deviations is reached at $M\;=\;10^{3}$, where both metrics stabilized with minimal variation beyond that point and correspond to the plateau of the coefficient of variation [40].

Based on this observation, the number of Monte Carlo simulations in the main analysis was fixed at $M\;=\;10^{3}$. This choice offers a good trade-off between precision and computational cost. The databases discussed in this paper are therefore generated using this value for M.

Since in the *m*-th Monte Carlo iteration a specific resonance *j* may either be identified multiple times or not identified at all, an acceptance interval on the natural frequency is defined based on the corresponding synchronous baseline reported in Table 1. In cases where multiple triplets fall within the same interval, the one with the highest MAC value relative to the baseline mode shape is selected for analysis. Conversely, if a resonance is not identified in a given iteration, no value is recorded for that case.

The number of successful identifications for resonance *j* over *M* total iterations is counted as $r_{j}$. This allows the definition of a success rate $\eta_{j}$, which quantifies the robustness of the SSI-COV algorithm at that specific level of non-deterministic sampling conditions (Equation (19)).

(19) $$\eta_{j}\;=\;\frac{r_{j}}{M}$$

Depending on whether the resonance *j* is successfully identified, each occurrence may contain valid values or remain unassigned. The success rate $\eta_{j}$ thus tracks the identification performance for each resonance across the entire simulation. Similarly to the confidence intervals, the success rate is parametrized over $\alpha_{x}$.

The analysis of the results is organized into two subsequent parts.

At first, the confidence intervals are defined under the sole effect of lack of synchronism, with the structure in its undamaged condition. A total of 51 datasets has been populated, each one for a specific value of $\alpha_{x}$ ranging from 0.01% to 10%, as listed in Table A1. The considered range is expressed relative to the nominal output data rate ($ODR_{nl}$) to reflect a realistic scenario in which non-deterministic sampling variability can only be approximately known. Expressing $\alpha_{x}$ in terms of the nominal $ODR_{nl}$ allows the evaluation of the sensor network’s performance limits for a specific SHM application. This, in turn, helps either to define the limitations of the current network or to guide the selection of more suitable sensor nodes. As a simple example, suppose that the framework applied to a specific SHM application shows that the estimated operational modal parameters remain robust even under large values of $\alpha_{x}$ for the sensor network deployed on the target structure. In that case, the use of low-cost solutions is justified and supported by the confidence intervals evaluated through the framework.

In the second phase, the confidence intervals have been estimated when the structure is subjected to the damage conditions d1, d2, and d3 (Table 2). This situation is combined with a non-deterministic sampling variability among the $\alpha_{x}\;=\;1.0\%,\;2.0\%$, and 3.0% values. This results in 69 additional datasets, listed in Table A2, which are used to assess the possibility to detect the presence of damage due to the intrinsic variability of the modal parameters related to non-deterministic sampling conditions. In this phase, the confidence intervals derived from the undamaged case serve as a reference for comparison.

### 5.1. Confidence Intervals Under Non-Deterministic Sampling for the Undamaged Structure

In this phase, the uncertainty on the operational modal parameters measured via confidence intervals $\Delta \omega_{j}/2\pi \left(\alpha_{x}\right)$, $\Delta \zeta_{j}\left(\alpha_{x}\right),$ and $\Delta \psi_{j}\left(\alpha_{x}\right)$, is attributed solely to the non-deterministic sampling variability level $\alpha_{x}$, which is the only variable parameter among the datasets (Table A1). The confidence intervals are also accompanied by the success rate $\eta_{j}\left(\alpha_{x}\right)$, which tracks the reliability of identifying each resonance.

Under otherwise identical conditions, datasets in Table A1 are produced for two nominal output data rates, $ODR_{nl}\;=\;500$ samples/s and $ODR_{nl}\;=\;833$ samples/s to assess the additional influence of a non-rational sampling interval (e.g., dt=1/833 s). These values can be easily found among commercially available digital MEMS modules.

**Figure 7:**
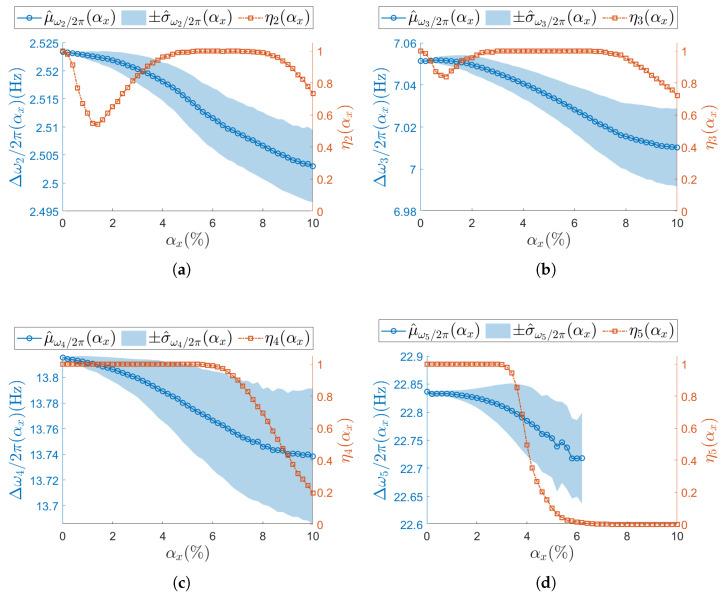
(**a**–**d**) Resonances from j=2 to j=5. Natural frequency (left vertical axis) for the undamaged structure and with each sensor node set to $ODR_{nl}\;=\;500$ samples/s. Success rate $\eta_{j}$ of the estimation process over *M* iterations at each $\alpha_{x}$ is shown on the right vertical axis. To improve visualization of the modal parameter, its sample mean $\hat{\mu }$ is illustrated as a circle (∘) and its sample standard deviation $\hat{\sigma }$ as shaded area. Concerning the success rate, the correspondent $\eta_{j}$ is reported as squared seed (□).

The results presented from this point onward (e.g., Figure 7) follow a consistent representation strategy. The confidence intervals of the target modal parameter are shown as a light-blue shaded area (color version only), while its mean values across different $\alpha_{x}$ levels are depicted by a solid blue line with circular markers indicating the tested $\alpha_{x}$ values. Additionally, the figure includes the evolution of the success rate $\eta_{j}\left(\alpha_{x}\right)$, displayed as a solid red line with red squared markers (color version only). Success rate values correspond to the right-hand axis, whereas the target modal parameter values refer to the left-hand axis.

**Figure 8:**
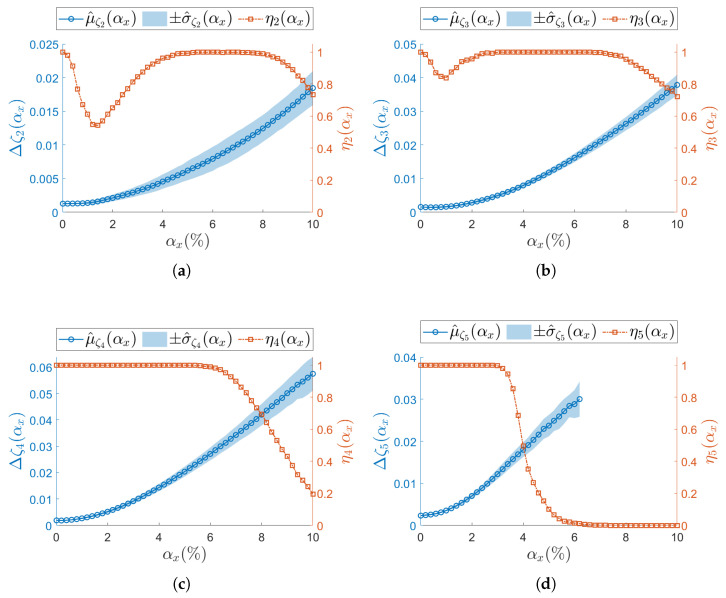
(**a**–**d**) Resonances from j=2 to j=5. Damping ratio (left vertical axis) for the undamaged structure and with each sensor node set to $ODR_{nl}\;=\;500$ samples/s. Success rate $\eta_{j}$ of the estimation process over *M* iterations at each $\alpha_{x}$ is shown on the right vertical axis. To improve visualization of the modal parameter, its sample mean $\hat{\mu }$ is illustrated as a circle (∘) and its sample standard deviation $\hat{\sigma }$ as a shaded area. Concerning the success rate, the correspondent $\eta_{j}$ is reported as squared seed (□).

**Figure 9:**
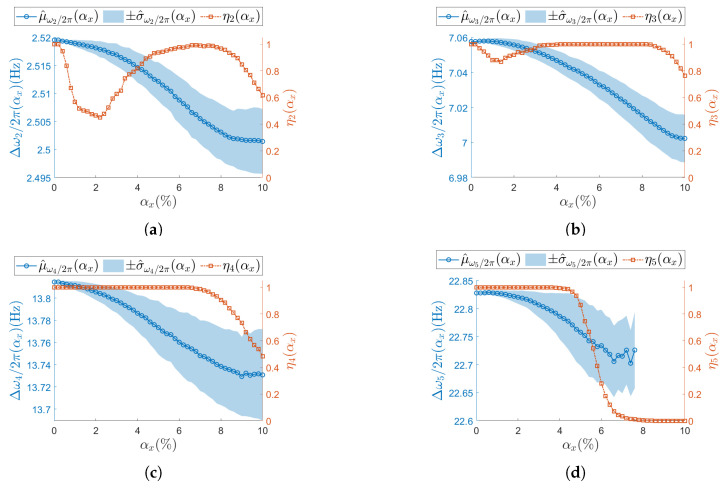
(**a**–**d**) Resonances from j=2 to j=5. Natural frequency (left vertical axis) for the undamaged structure and with each sensor node set to $ODR_{nl}\;=\;833$ samples/s. Success rate $\eta_{j}$ of the estimation process over *M* iterations at each $\alpha_{x}$ is shown on the right vertical axis. To improve visualization of the modal parameter, its sample mean $\hat{\mu }$ is illustrated as a circle (∘) and its sample standard deviation $\hat{\sigma }$ as a shaded area. Concerning the success rate, the correspondent $\eta_{j}$ is reported as squared seed (□).

**Figure 10:**
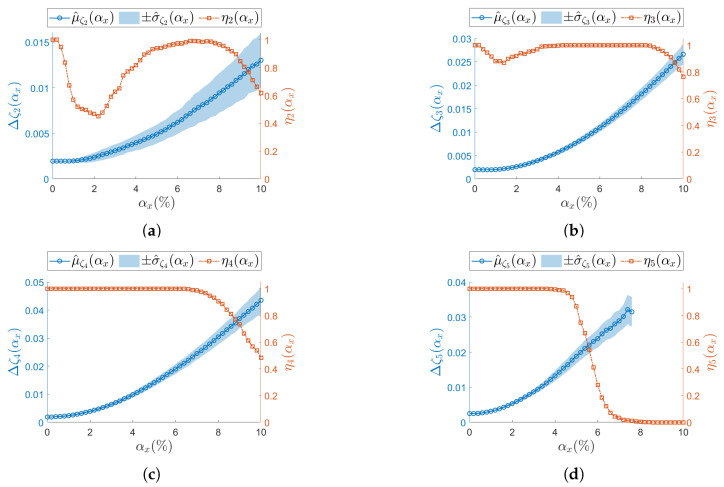
(**a**–**d**) Resonances from j=2 to j=5. Damping ratio (left vertical axis) for the undamaged structure and with each sensor node set to $ODR_{nl}\;=\;833$ samples/s. Success rate $\eta_{j}$ of the estimation process over *M* iterations at each $\alpha_{x}$ is shown on the right vertical axis. To improve visualization of the modal parameter, its sample mean $\hat{\mu }$ is illustrated as a circle (∘) and its sample standard deviation $\hat{\sigma }$ as shaded area. Concerning the success rate, the correspondent $\eta_{j}$ is reported as squared seed (□).

Figure 7, Figure 8, Figure 9 and Figure 10 present the results of the analysis for the scalar operational modal parameters for the two simulated nominal ODRs, namely $ODR_{nl}\;=\;500$ samples/s (Figure 7 and Figure 8) and $ODR_{nl}\;=\;833$ samples/s (Figure 9 and Figure 10). Specifically, Figure 7 and Figure 9 account for the results on the natural frequencies. Correspondingly, Figure 8 and Figure 10 report the associated trends observed for the modal damping ratios. Since the success rate $\eta_{j}$ reflects the overall reliability of the identification process for the triplet of operational modal parameters $\left(\omega_{j}/2\pi ,\zeta_{j},\psi_{j}\right)$, it is shown in the plots for both natural frequencies and damping ratios. For instance, in the case of $ODR_{nl}\;=\;500$ samples/s, the results for resonance j=2 are presented in Figure 7a and Figure 8a, and thus the corresponding $\eta_{j}$ is identical in both plots.

For increasing $\alpha_{x}$, different $\eta_{j}$ (Equation (19)) trends are observed across resonances, meaning that the number of times the *j*-th resonance is identified by SSI-COV is not uniform: focusing on low-frequency resonances, at both j=2 (Figure 7a, Figure 8a and Figure 9a) and j=3 (Figure 7b, Figure 8b and Figure 9b), $\eta_{j}$ drop to a minimum between $\alpha_{x}=1.4\%$ and $\alpha_{x}=2.0\%$. Moving at higher frequencies, at resonance j=4 (Figure 7c, Figure 8c and Figure 9c), no local minimum in $\eta_{j}$ is observed until the non-deterministic sampling variability becomes significant. A critical condition is noticed at the last resonance value j=5 (Figure 7d, Figure 8d and Figure 9d), where the $\eta_{j}$ parameter drops to zero. This classifies j=5 as the resonance most sensitive to $\alpha_{x}$. The specific values assumed by $\eta_{j}$ are different for $ODR_{nl}=500$ samples/s and $ODR_{nl}=833$ samples/s; nevertheless, the same trend is recognizable (e.g., for resonance j=5, see Figure 7d, Figure 8d and Figure 9d).

Regarding the confidence intervals $\Delta \omega_{j}/2\pi \left(\alpha_{x}\right)$ (Equation (16)), as $\alpha_{x}$ increases, the sample standard deviation ${\hat{\sigma }}_{\omega_{j}/2\pi }\left(\alpha_{x}\right)$ also increases. At the same time, the sample mean ${\hat{\mu }}_{\omega_{j}/2\pi }\left(\alpha_{x}\right)$ shows the opposite trend. Therefore, a larger non-deterministic sampling variability brings a larger underestimation in the natural frequency. This trend is confirmed at both $ODR_{nl}$ and in all resonances: j=2 (Figure 7a, Figure 8a and Figure 9a), j=3 (Figure 7b, Figure 8b and Figure 9b), j=4 (Figure 7c, Figure 8c and Figure 9c), and j=5 (Figure 7d, Figure 8d and Figure 9d). At resonance j=5, consistently with the previous analysis on $\eta_{j}$, confidence intervals lose statistical significance when $\eta_{j}$ drops to zero, and they are completely unavailable once after $\eta_{5}$ has dropped to zero.

The analysis on the confidence intervals of damping ratio $\Delta \zeta_{j}\left(\alpha_{x}\right)$ (Equation (17)) is consistently reported in Figure 8 ($ODR_{nl}\;=\;500$ samples/s) and in Figure 10 ($ODR_{nl}\;=\;833$ samples/s). All resonances show that both the sampling mean ${\hat{\mu }}_{\zeta_{j}}$ and the sampling standard deviation ${\hat{\mu }}_{\zeta_{j}}$ increase for increasing $\alpha_{x}$—see, as example, resonance j=2 in Figure 8a, Figure 9a and Figure 10a. Again, those trends are observed at both the considered $ODR_{nl}$.

**Figure 11:**
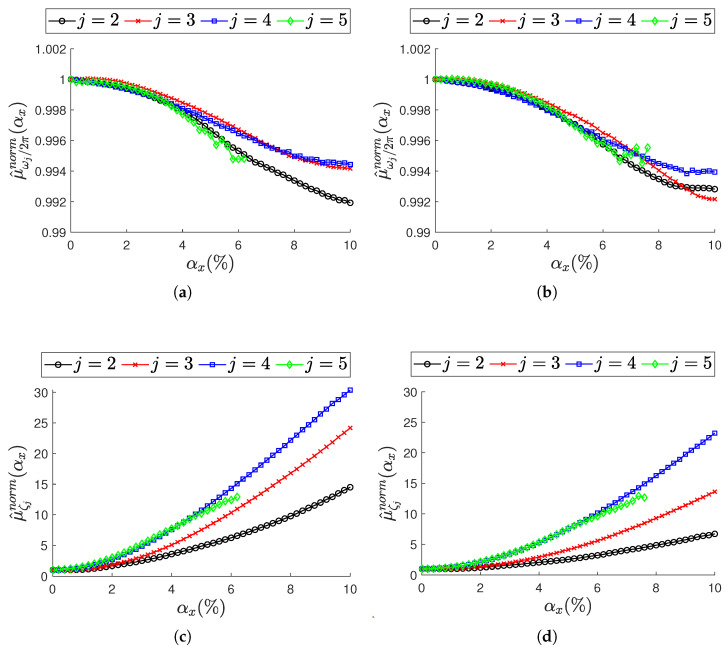
Effect of non-constant sampling variability level $\alpha_{x}$ across resonances j={2,3,4,5}. Top row (**a**,**b**): natural frequencies in terms of normalized mean value (Equation (20)). Bottom row (**c**,**d**): damping ratios in terms of normalized mean value (Equation (21)). (**a**,**c**) Sensor nodes are set to $ODR_{nl}\;=\;500$ samples/s. (**b**,**d**) sensor nodes are set to $ODR_{nl}\;=\;833$ samples/s. In each plot, trends are reported for increasing level of $\alpha_{x}$.

To summarize the analysis on the scalar modal parameters $\omega_{j}/2\pi$ and $\zeta_{j}$, a comparison is provided in Figure 11 in terms of the mean value across resonances. On the left, Figure 11a–c corresponds to $ODR_{nl}=500$ samples/s; on the right, Figure 11b–d refer to $ODR_{nl}=833$ samples/s. To make a comparison across resonances, the mean value is normalized over the most synchronous database (i.e., when $\alpha_{x}=0.01\%$), as shown in Equation (20) for $\omega_{j}/2\pi$ and in Equation (21) for $\zeta_{j}$.

(20) $$\begin{array}{cc} {\hat{\mu }}_{\omega_{j}/2\pi }^{norm}\left(\alpha_{x}\right) & =\frac{{\hat{\mu }}_{\omega_{j}/2\pi }\left(\alpha_{x}\right)}{{\hat{\mu }}_{\omega_{j}/2\pi }\left(\alpha_{x}=0.01\%\right)} \end{array}$$

(21) $$\begin{array}{cc} {\hat{\mu }}_{\zeta_{j}}^{norm}\left(\alpha_{x}\right) & =\frac{{\hat{\mu }}_{\zeta_{j}}\left(\alpha_{x}\right)}{{\hat{\mu }}_{\zeta_{j}}\left(\alpha_{x}=0.01\%\right)} \end{array}$$

Considering all resonances, some important remarks for increasing non-deterministic sampling variability levels $\alpha_{x}$ can be pointed out: the natural frequencies (Figure 11a,b) are underestimated, while the damping ratios (Figure 11c,d) are overestimated. The normalized mean value highlights the natural frequencies to be more stable, while the damping ratios are subjected to larger variation. At resonance j=5, where the identification of the modal parameter process becomes critical, a noisy trend is observed at natural frequencies (Figure 11a,b): this can reasonably be attributed to the loss of statistical significance of the mean value over the *M* iterations, as previously noted.

**Figure 12:**
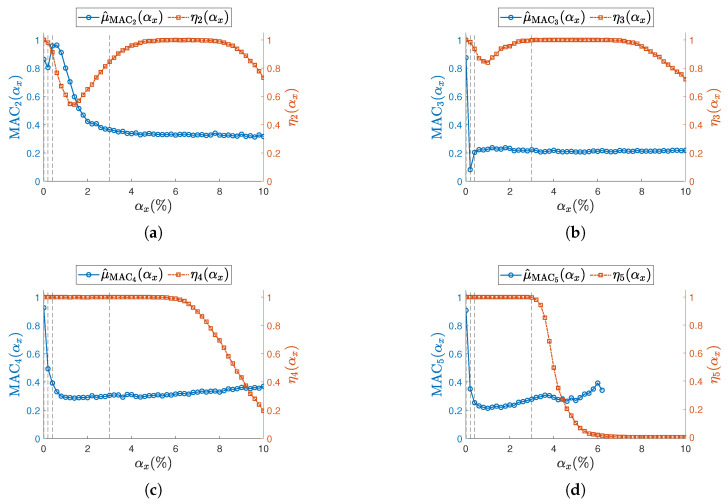
(**a**–**d**) Resonances from j=2 to j=5. Sampling mean value of MAC (left vertical axis) for the undamaged structure and with each sensor node set to $ODR_{nl}=500$ samples/s. Success rate $\eta_{j}$ at each $\alpha_{x}$ is shown on the right vertical axis. To improve visualization of the MAC, its sample mean is illustrated as a circle (∘). Concerning the success rate, the corresponding $\eta_{j}$ is reported as squared seed (□). Vertical dashed lines (- - -) indicate the selected $\alpha_{x}$ reported in Figure 14.

**Figure 13:**
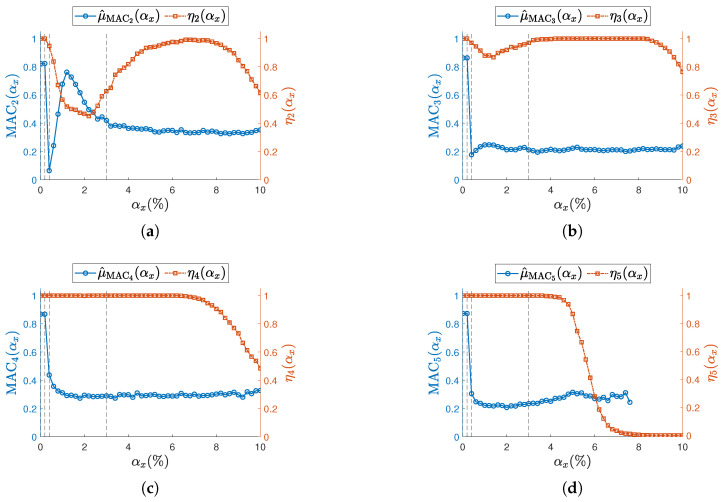
(**a**–**d**) Resonances from j=2 to j=5. Sampling mean value of MAC (left vertical axis) for the undamaged structure and with each sensor node set to $ODR_{nl}=833$ samples/s. Success rate $\eta_{j}$ at each $\alpha_{x}$ is shown on the right vertical axis. To improve visualization of the MAC, its sample mean is illustrated as a circle (∘). Concerning the success rate, the corresponding $\eta_{j}$ is reported as squared seed (□). Vertical dashed lines (- - -) indicate the selected $\alpha_{x}$ reported in Figure 15.

The confidence interval for the last modal parameter—namely, the mode shapes $\Delta \psi_{j}\left(\alpha_{x}\right)$—is handled slightly differently, since $\omega_{j}/2\pi$ and $\zeta_{j}$ are scalars, while $\psi_{j}$ is a vector of *S* elements. To maintain a consistent representation with natural frequencies and damping ratios, the Modal Assurance Criterion (MAC) is used and reported in Figure 12 and Figure 13. MAC is computed for each dataset and each level of sampling variability $\alpha_{x}$, by comparing $\psi_{j}\left(\alpha_{x}\right)$ with the corresponding synchronous case $\psi_{j}\left(\alpha_{x}\;=\;0\right)$. The sample mean of ${\mathrm{MAC}}_{j}$ is then plotted in the aforementioned figures. Standard deviations are not included, as MAC is a normalized metric—intervals above 1 would be meaningless.

Across all resonances and for both $ODR_{nl}$ values, the MAC consistently shows a sharp decline even at the initial levels of sampling variability. This trend aligns with previous findings in the literature [12,13], which attribute such degradation to the loss of physical phase consistency between sensor nodes.

**Figure 14:**
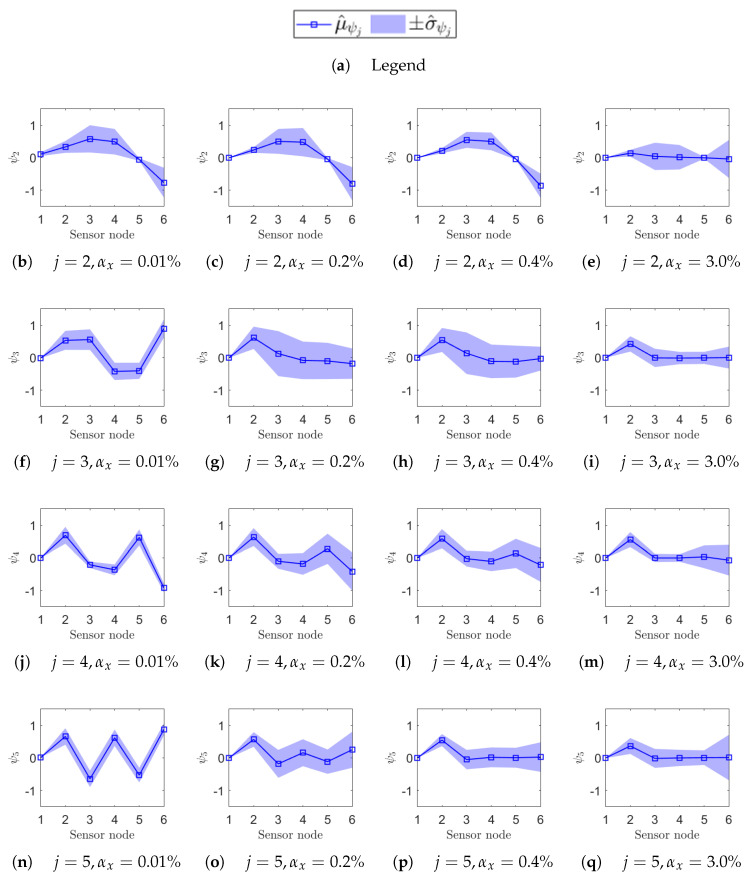
Uncertainty on the estimation of the mode shape $\psi_{j}$ of each *j*-th resonance (rows) due to a given non-deterministic sampling variability $\alpha_{x}$ (columns). The structure is undamaged, and each sensor node is set to $ODR_{nl}\;=\;500$ samples/s. In each plot, the confidence interval $\Delta \psi_{j}\;=\;{\hat{\mu }}_{\psi_{j}}+{\hat{\sigma }}_{\psi_{j}}$ over *M* iterations at that $\alpha_{x}$ is reported on the sensor node location along the beam axis: ${\hat{\mu }}_{\psi_{j}}$ as a squared seed (□) and ${\hat{\sigma }}_{\psi_{j}}$ as a shaded area.

**Figure 15:**
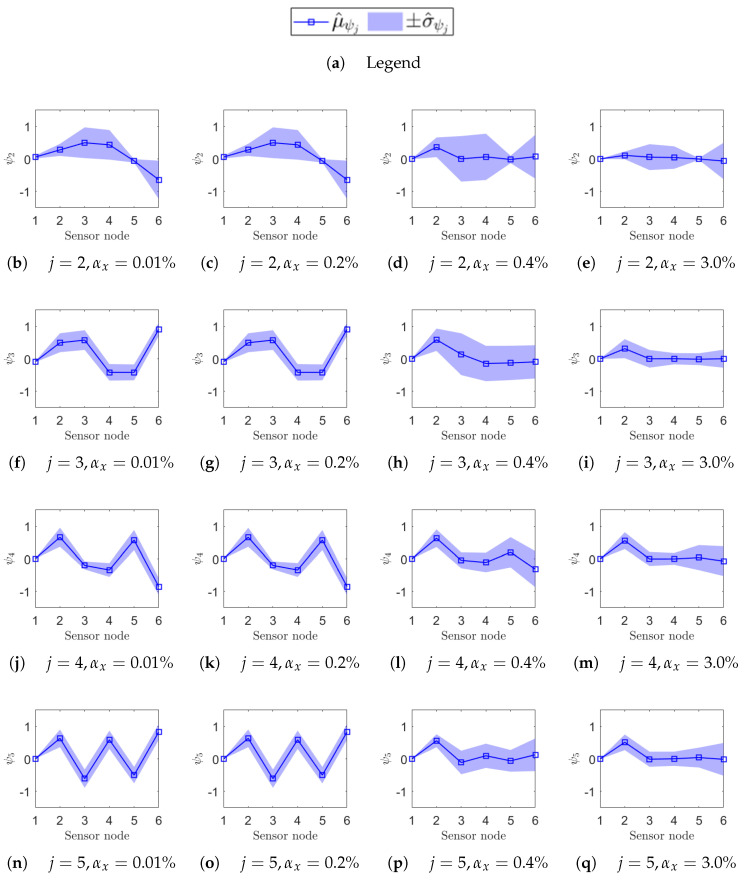
Uncertainty on the estimation of the mode shape $\psi_{j}$ of each *j*-th resonance (on rows) due to a given non-deterministic sampling variability $\alpha_{x}$ (on columns). The structure is undamaged, and each sensor node is set to $ODR_{nl}\;=\;833$ samples/s. In each plot, the confidence interval $\Delta \psi_{j}\;=\;{\hat{\mu }}_{\psi_{j}}+{\hat{\sigma }}_{\psi_{j}}$ over *M* iterations at that $\alpha_{x}$ is reported on the sensor node location along the beam axis: ${\hat{\mu }}_{\psi_{j}}$ as a squared seed (□) and ${\hat{\sigma }}_{\psi_{j}}$ as a shaded area.

To highlight the high sensitivity of mode shapes to non-deterministic sampling, selected confidence intervals for $\Delta \psi_{j}\left(\alpha_{x}\right)$, defined in Equation (18), are reported for specific values $\alpha_{x}\;=\;\left\{0.01\%,0.2\%,0.4\%,3\%\right\}$, indicated by vertical dashed lines in Figure 12 and Figure 13. The first three values correspond to the range where phase relationships begin to degrade, while $\alpha_{x}\;=\;3\%$ lies within the early part of the MAC plateau. In Figure 14 ($ODR_{nl}=500$ samples/s) and Figure 15 ($ODR_{nl}=833$ samples/s), a grid of plots is arranged so that a row refers to a specific *j*-th resonance, and a column refers to a specific level of non-deterministic sampling variability $\alpha_{x}$. Each plot is arranged so that the physical phase relationship between sensorized DOFs can be compared with those carried out in the eigenvalue analysis in Figure 3, which represents the ideal case. For example, Figure 14f–i are displayed on the same rows and refer to resonance j=3, to be cross-checked with Figure 3c. For the sake of clearness, confidence intervals are shown for a few selected values of non-deterministic sampling variability $\alpha_{x}$, arranged in columns. As already mentioned, previous studies [12,18] pointed out that even a tiny time misalignment is sufficient to lose the physical phase relationship between sensorized DOFs.

At the most synchronous datasets $\alpha_{x}=0.01\%$, mode shapes reasonably resemble those from the eigenvalue analysis (MAC values around 0.8). Moving to larger but contained levels of $\alpha_{x}=\left\{0.2\%,0.4\%\right\}\%$, mode shapes behave slightly differently under the two nominal sampling conditions of 500 samples/s and 833 samples/s; that is confirmed by cross-checking the correspondent MAC. For instance, for $\alpha_{x}=0.2\%$, the physical phase relationship at resonance j=3 is lost in case of $ODR_{nl}=500$ samples/s (Figure 14g), but is retained in case of $ODR_{nl}=833$ samples/s (Figure 15g); conversely, the physical phase relationship at resonance j=2 for $\alpha_{x}=0.4\%$ is preserved at $ODR_{nl}=500$ samples/s (Figure 14c) but becomes unreliable at $ODR_{nl}=833$ samples/s (Figure 15c). As expected, the non-deterministic sampling variability of $\alpha_{x}=3\%$ reported in the last column makes the mode shapes completely unrecognizable and unusable.

### 5.2. Confidence Intervals in Presence of a Structural Alteration Under Non-Deterministic Sampling

The previous section established the confidence intervals for operational modal parameters (Equations (16)–(18)) for the target system in the undamaged condition. That baseline now serves as a reference for evaluating the reliability in detecting potential damage to the target structure under non-deterministic sampling condition.

The baseline analysis encouraged focusing on the two most stable parameters under non-deterministic sampling: the natural frequency and modal damping ratio, with $\omega_{j}/2\pi$ being less susceptible than $\zeta_{j}$ to ODR variability. Given the high sensitivity of the physical phase relationship between sensorized DOFs to even a tiny sampling oscillation from the nominal value $ODR_{nl}$, the use of mode shapes $\psi_{j}$ as damage-sensitive features is not recommended and is therefore excluded from this part of the analysis.

Moreover, the comparison presented earlier between the two ODR values, i.e., 500 samples/s and 833 samples/s, showed very similar trends. Hence, this second phase includes results for the $ODR_{nl}\;=\;500$ samples/s case only.

The estimated natural frequencies and damping ratios refer to three levels of non-deterministic sampling variability, specifically $\alpha_{x}$ = {1.0%, 2.0%, 3.0%}; these levels are selected to represent three different conditions of increasing sampling variability (the confidence intervals from the baseline are available for these conditions).

These levels of $\alpha_{x}$ are tested for the three damage conditions (one at a time), d1, d2, and d3. The damage conditions refer to the stiffness reduction severity levels defined in Table 2. This results in a total of 69 simulated datasets, as listed in Table A2. In this notation, confidence intervals from the *d*-th damaged scenario are indicated as $\Delta {\omega_{j}/2\pi }^{d}$ for natural frequencies and $\Delta {\zeta_{j}}^{d}$ for damping ratios, where the superscript *d* denotes the damage case.

For localized damage scenarios, the analysis focuses on the most critical resonance, namely j=2 for d1, and j=4 for d2.

The previous analysis on the undamaged structures shows that, for increasing non-deterministic sampling variability level $\alpha_{x}$, the confidence interval $\Delta \omega_{j}/2\pi$ decreases in terms of mean value ${\hat{\mu }}_{\omega_{j}/2\pi }$ and becomes wider in terms of standard deviation ${\hat{\sigma }}_{\omega_{j}/2\pi }$ (e.g., see Figure 7). At the same time, when the structure undergoes a stiffness reduction (see Equation (3)), the natural frequencies are expected to decrease with increasing damage severity.

**Figure 16:**
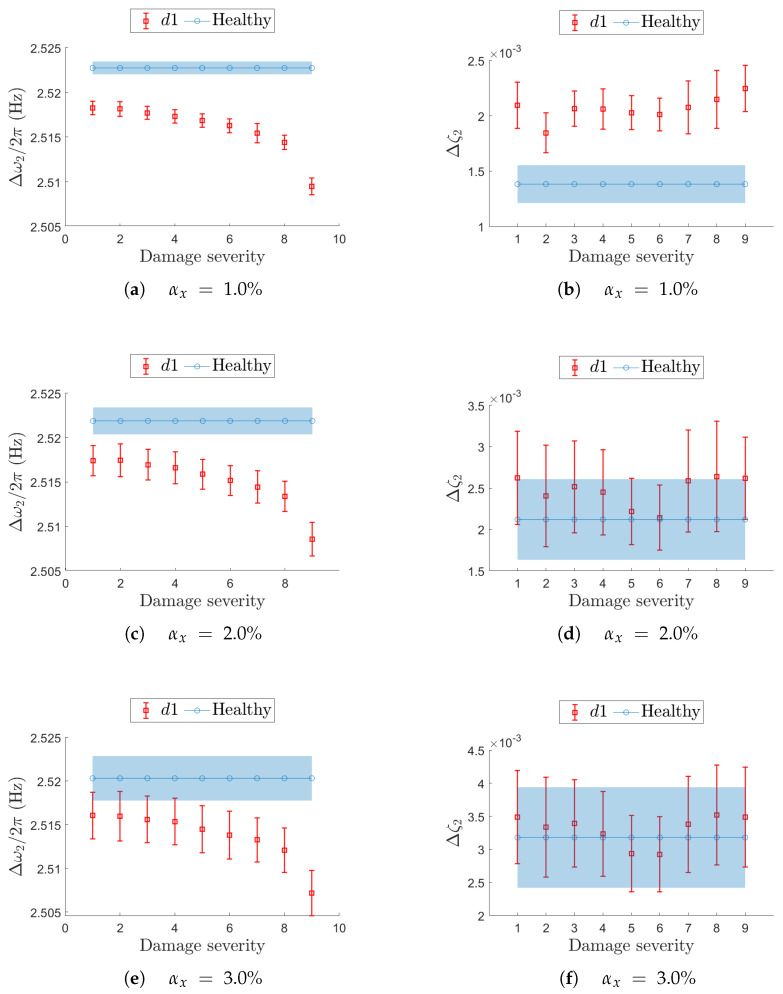
Effect of a local stiffness reduction in d1 on resonance j=2 in terms of confidence intervals on the natural frequency (left-hand column) and damping ratio (right-hand column). On each row, a different value for the non-deterministic sampling variability $\alpha_{x}$ is used. In each plot, the evolution of the confidence interval is reported for increasing damage severity and compared with the corresponding confidence interval from the undamaged (healthy) structure.

**Figure 17:**
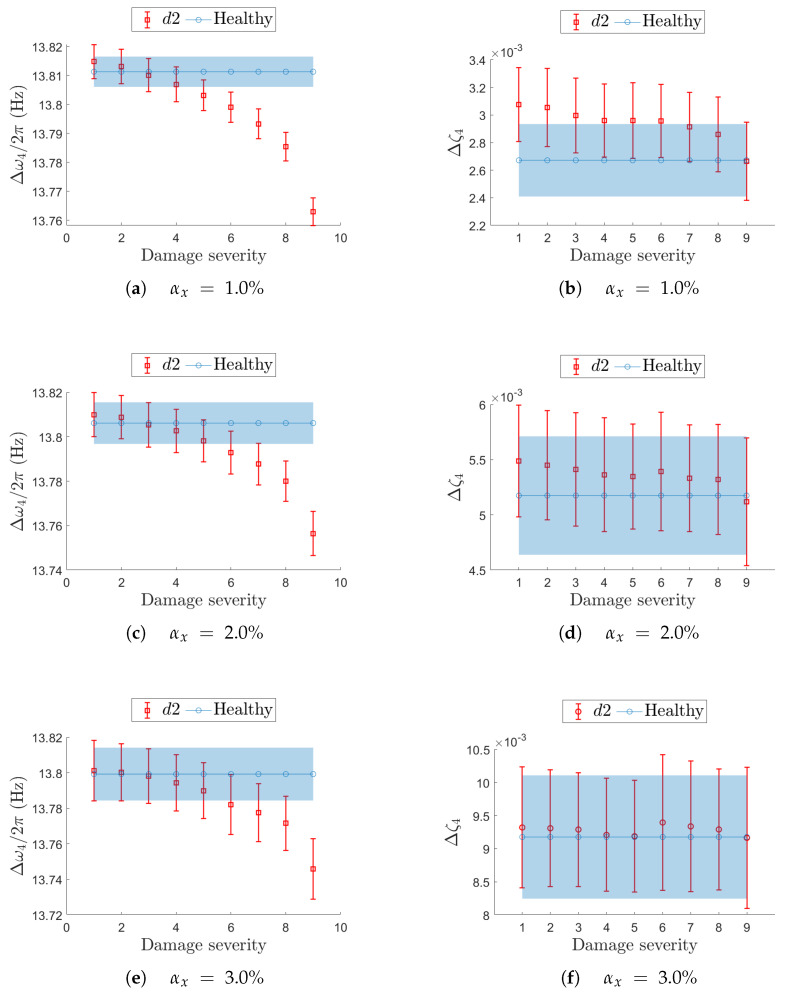
Effect of a local stiffness reduction in d2 on resonance j=4 in terms of confidence intervals on the natural frequency (left-hand column) and damping ratio (right-hand column). On each row, a different value for the non-deterministic sampling variability $\alpha_{x}$ is used. In each plot, the evolution of the confidence interval is reported for increasing damage severity and compared with the corresponding confidence interval from the undamaged (healthy) structure.

Results presented in Figure 16 and Figure 17 follow the notation described below. The filled light-blue area and the solid blue line with circular markers (in the color version) represent the confidence interval and the mean value of the target modal parameter in the undamaged condition (i.e., constant reference values). The red bars, which vary with damage severity, indicate the confidence intervals estimated for each specific damage scenario tested at the different $\alpha_{x}$ values.

Damage location d1 for resonance j=2 is reported in fig:d1mode1. The left columns show the natural frequency, while the right columns report the corresponding damping ratio. On the horizontal axis, the damage severity (i.e., stiffness reduction) is reported for increasing values. The expected sensitivity of natural frequency (Figure 16a,c,e) to a stiffness reduction is reported in terms of confidence intervals, $\Delta {\omega_{j}/2\pi }^{d1}$, as well as the baseline from the undamaged condition, $\Delta \omega_{j}/2\pi$: at $\alpha_{x}\;=\;3.0\%$ (Figure 16e), $\Delta {\omega_{j}/2\pi }^{d1}$ and $\Delta \omega_{j}/2\pi$ are overlapped up to damage severity equal to 3. This suggests that early-stage damage is not clearly detectable from resonance j=2 under such a non-deterministic sampling variability level, and the damage becomes distinguishable only at higher damage severity. Conversely, damping estimation is more affected by the non-deterministic sampling than the stiffness reduction: at $\alpha_{x}=3\%$ (Figure 16f), the confidence intervals are completely overlapped on those from the baseline, even at the most severe stiffness reduction.

Damage case d2 is depicted in Figure 17 for resonance j=4. The fact that non-deterministic sampling variability outweighs the impact of a stiffness reduction is visible even at lower level $\alpha_{x}\;=\;1\%$, for both the natural frequency (Figure 17a) and the damping ratio (Figure 17b). The stability of natural frequency with respect to sampling variability confirms that sensor networks with limited sampling uncertainty are better suited to detect damage than those affected by higher sampling uncertainty. Even in this second damage case d2, the damping ratio is too heavily influenced by uncertain sampling to serve as a reliable damage-sensitive feature.

**Figure 18:**
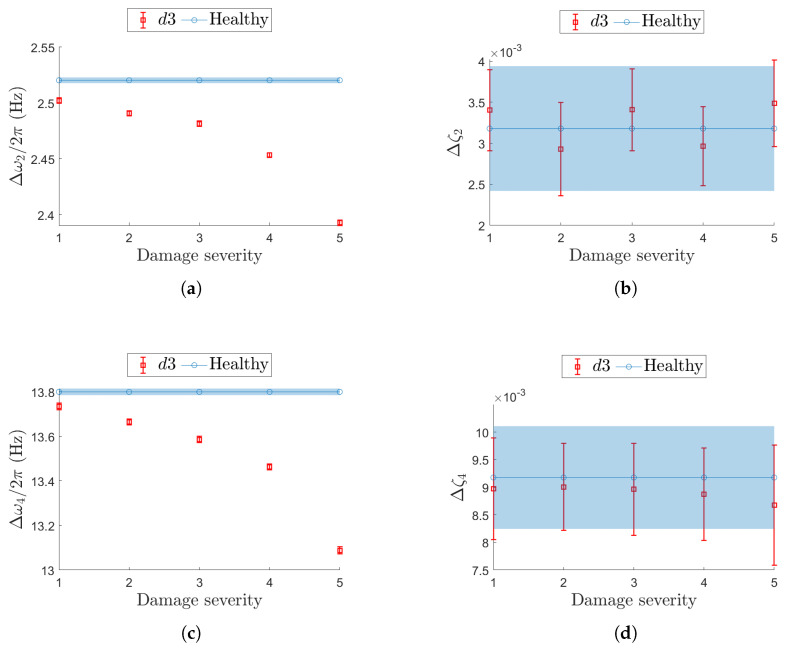
Effect of a global stiffness reduction (d3) on resonance j=2 (top row (**a**,**b**)) and j=4 (bottom row (**c**,**d**)) in terms of confidence intervals on the natural frequency (left-hand column (**a**,**c**)) and damping ratio (right-hand column (**b**,**d**)). The same value for the non-deterministic sampling variability, $\alpha_{x}=3\%$, is used. In each plot, the evolution of the confidence interval is reported for increasing damage severity and compared with the corresponding confidence interval from the undamaged (healthy) structure.

Damage case d3 considers a uniform, distributed reduction in stiffness across the entire beam and is analyzed across resonances j=2 and j=4, coherently with the analysis on d1 and d2. In Figure 18, the confidence intervals for the most critical non-deterministic sampling variability level $\alpha_{x}\;=\;3.0\%$ are compared with the baseline. As the d3 case represents an extreme scenario, the results confirm that natural frequency remains the most robust damage-sensitive parameter under sampling uncertainty (Figure 18a,c). Conversely, damping appears to be more influenced by sampling inconsistencies than by the structural alteration itself (Figure 18b,d).

## 6. Conclusions

Digital sensor nodes remain an appealing choice for SHM system design, as the use of digital MEMS-based accelerometers and MCUs enables network scalability, integration in both wired and wireless configurations, hardware redundancy, and onboard data processing. However, the presence of multiple independent clock sources—one per component—can introduce non-deterministic sampling variability in the absence of a shared, deterministic time reference. This becomes critical in OMA-based SHM applications, where phase inconsistency across sensor nodes can bias damage-sensitive features.

To address the link between node-level sampling uncertainty and its effect on operational modal parameters, a Monte Carlo framework was developed. It assumes invariance in EOVs, firmware configuration, sensor placement, and the identification algorithm. Intra- and inter-node sampling variability are modeled via a statistical distribution calibrated with vendor-provided information, from nominal ODR ($ODR_{nl}$) to actual sampling rate variability ($\alpha_{x}$).

The framework was applied to a numerical system with well-separated modes, using a standard firmware setup lacking advanced resynchronization strategies. Results align with findings by Lei [12] and Bernal [13]: natural frequencies are generally robust to lack of synchronization, whereas even minimal misalignment is sufficient to degrade mode shapes, making them unreliable for damage detection. Damping ratios also show increased sensitivity, consistent with prior observations [33].

Importantly, this study demonstrates that natural frequencies—often viewed as robust indicators—can lose reliability in damaged configurations. Confidence intervals derived under healthy conditions may not extend to altered states, limiting their effectiveness for SHM.

The framework offers a practical tool for addressing sampling variability in OMA applications and encourages a more comprehensive engineering approach. Additional interdependent sources of uncertainty—such as modal complexity, EOVs, and SNR—should be considered alongside sampling effects. Future improvements may include integrating experimental insights on actual ODR fluctuations and using the framework as a benchmark to compare modal identification strategies under realistic variability conditions.

A key limitation of the method is its computational cost. Monte Carlo simulations require significant resources to generate statistically meaningful datasets. Alternative sampling strategies with improved efficiency could be explored to enhance the framework’s practicality for large-scale or real-time applications.

## Figures and Tables

**Table 1:** Natural frequencies from the eigenvalue analysis for the undamaged structure.

*j*	1	2	3	4	5	6
$\omega_{j}/2\pi$ (Hz)	0.402	2.520	7.054	13.820	22.841	34.117

**Table 2:** Young’s modulus reduction coefficient, δ, arranged for increasing severity. Damage location is reported the leftmost column, as indicated by arrow (↴). Severity levels are reported in the top row, as indicated by arrow (→).

Location ↴ Severity →	1	2	3	4	5	6	7	8	9
d1, d2	0.05	0.10	0.20	0.30	0.40	0.50	0.60	0.70	0.90
d3	0.01	0.02	0.03	0.05	0.10	-	-	-	-

**Table 3:** Values for the SSI-COV setup parameters.

τ	$n_{\min}$	$n_{\max}$	$\epsilon_{\omega /2\pi }$	$\epsilon_{\zeta }$	$\epsilon_{\mathrm{MAC}}$	$\epsilon_{\mathrm{cluster}}$
2 (s)	10	30	0.01	0.01	0.01	4 (Hz)

## Data Availability

The raw data supporting the conclusions of this article will be made available by the authors on request.

## Abbreviations

The following abbreviations are used in this manuscript.

ACF	Auto-Covariance Function
ADC	Analog-to-Digital Converter
ARMAV	Auto-Regressive Moving Average Vector
DOF	Degree Of Freedom
EOV	Environmental and Operational Variable
FE	Finite Element
GPS	Global Positioning System
HPC	High Performance Computing
ISPU	Internal Signal Processing Unit
LTI	Linear and Time-Invariant
MAC	Modal Assurance Criterion
MCU	Micro-Controller Unit
MEMS	Micro Electro-Mechanical System
NTP	Network Time Protocol
ODR	Output Data Rate
OMA	Operational Modal Analysis
SHM	Structural Health Monitoring
SSI	Stochastic Subspace Identification
SSI-COV	COVariance-driven, SSI
WSN	Wireless Sensor Network

## List of Symbols

Symbols and their definitions used throughout the manuscript.

**Symbol**	**Meaning**
*j*	Indicator of the resonance of the structure
$\omega_{j}/2\pi$	Natural frequency of the j-th resonance
$\zeta_{j}$	Modal damping ratio of the j-th resonance
$\phi_{j}$	Mode shape vector from eigenvalue analysis of the j-th resonance
$\psi_{j}$	Mode shape vector at sensor locations, so that $\psi_{j}\subseteq \phi_{j}$
*S*	Number of observed degrees of freedom (DOFs)
g(t)	Stochastic ambient excitation
x(t)	Trial solution vector of the FE model
*E*	Young’s modulus of the beam material
ρ	Mass density of the beam material
δ	Stiffness reduction coefficient
$d_{1}$, $d_{2}$, $d_{3}$	Predefined structural damage scenarios in the FE model: $d_{1}$ and $d_{2}$ are localized stiffness reductions, $d_{3}$ is distributed
$E_{d}$	Young’s modulus of damaged elements
D	Damping matrix
M	Mass matrix
K	Stiffness matrix
γ	Mass-proportional damping coefficient
β	Stiffness-proportional damping coefficient
$y_{c,s}$	Data chunk retrieved from the FIFO buffer of the *s*-th sensor node during chunk *c*
$y_{s}$	Synchronous acceleration data record at sensor node *s*
Y	Matrix of synchronous acceleration data from all sensor nodes over time
*s*	Sensor node index (s=1,…,S)
*T*	Total duration of the data record
τ	Time lag parameter in SSI-COV
A	State matrix in SSI-COV
C	Output matrix in SSI-COV
x[k]	State vector at discrete time *k*
y[k]	Output vector at discrete time *k*
w[k]	Disturbance vector at discrete time *k*
v[k]	Measurement noise vector at discrete time *k*
$n_{\min},n_{\max}$	Minimum and maximum model orders for SSI-COV
$\epsilon_{\omega /2\pi }$	Stability threshold on natural frequency for SSI-COV
$\epsilon_{\zeta }$	Stability threshold on damping ratio for SSI-COV
$\epsilon_{\mathrm{MAC}}$	Threshold for modal assurance criterion for SSI-COV
$\epsilon_{\mathrm{cluster}}$	Tolerance for clustering similar poles in Hz for SSI-COV
$f_{S}$	Nominal sampling rate
dt	Nominal sampling interval
$\alpha_{x}$	Coefficient of variation of the sampling rate
$ODR_{nl}$	Nominal ODR of the *digital* sensor node (i.e., expected constant $f_{S}$)
$x_{c,s}$	Random variable representing ODR of chunk *c* for sensor node *s*
$\mu_{x}=ODR_{nl}$	Mean value of the Gaussian ODR distribution $N(x_{c,s}|\mu_{x},\sigma_{x}^{2})$
$\sigma_{x}^{2}=\alpha_{x}^{2}ODR_{nl}^{2}$	Variance of the Gaussian ODR distribution $N(x_{c,s}|\mu_{x},\sigma_{x}^{2})$
$t_{c,s}$	Time resolution (sampling interval) for chunk *c* of sensor *s*
${\hat{t}}_{s}$	Asynchronous time basis generated for sensor node *s* under non-deterministic sampling
${\hat{y}}_{s}$	Interpolated acceleration record for sensor node *s* corresponding to ${\hat{t}}_{s}$
$\hat{Y}$	Matrix of interpolated (non-synchronous) acceleration signals used as input to SSI-COV
$K_{c}$	Number of samples in each data chunk
*M*	Number of Monte Carlo simulations
$r_{j}$	Number of successful identifications of the *j*-th resonance across all *M* iterations
$\eta_{j}$	Success rate of identifying *j*-th resonance over *M* iterations
$\Delta \omega_{j}/2\pi \left(\alpha_{x}\right)$	Confidence interval of *j*-th frequency under sampling variability
$\Delta \zeta_{j}\left(\alpha_{x}\right)$	Confidence interval of *j*-th damping ratio under sampling variability
$\Delta \psi_{j}\left(\alpha_{x}\right)$	Confidence interval of *j*-th mode shape under sampling variability
${\hat{\mu }}_{\left(.\right)}\left(\alpha_{x}\right)$	Sample mean of a modal parameter (.) across *M* simulations at given $\alpha_{x}$
${\hat{\sigma }}_{\left(.\right)}\left(\alpha_{x}\right)$	Sample standard deviation of a modal parameter (.) across *M* simulations at given $\alpha_{x}$
${\hat{\mu }}_{\left(.\right)}^{\mathrm{norm}}\left(\alpha_{x}\right)$	Normalized sample mean of a modal parameter (.), referenced to the most synchronous case (e.g., $\alpha_{x}\;=\;0.01\%$)
$\Delta {\left(.\right)}^{d}$	Confidence interval of a modal parameter (.) under a given structural damage condition ($d_{1}$, $d_{2}$, or $d_{3}$)

## Appendix A. Pseudo-Code of a Single Monte Carlo Iteration

**Algorithm 1:** Generation of non-synchronous records for SSI-COV

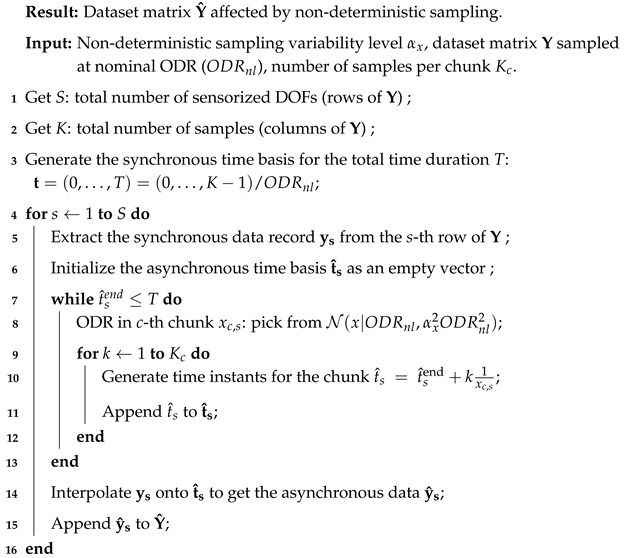

## Appendix B. Simulated Datasets

**Table A1:** List of datasets for the undamaged case, numbered for increasing $\alpha_{x}$.

$\alpha_{x}\left(\%\right)$	**0.01**	**0.2**	**0.4**	**0.6**	**0.8**	**1.0**	**1.2**	**1.4**	**1.6**	**1.8**	**2.0**
n.	1	2	3	4	5	6	7	8	9	10	11
$\alpha_{x}\left(\%\right)$	**2.2**	**2.4**	**2.6**	**2.8**	**3.0**	**3.2**	**3.4**	**3.6**	**3.8**	**4.0**	**4.2**
n.	12	13	14	15	16	17	18	19	20	21	22
$\alpha_{x}\left(\%\right)$	**4.4**	**4.6**	**4.8**	**5.0**	**5.2**	**5.4**	**5.6**	**5.8**	**6.0**	**6.2**	**6.4**
n.	23	24	25	26	27	28	29	30	31	32	33
$\alpha_{x}\left(\%\right)$	**6.6**	**6.8**	**7.0**	**7.2**	**7.4**	**7.6**	**7.8**	**8.0**	**8.2**	**8.4**	**8.6**
n.	34	35	36	37	38	39	40	41	42	43	44
$\alpha_{x}\left(\%\right)$	**8.8**	**9.0**	**9.2**	**9.4**	**9.6**	**9.8**	**10.0**		-	-	-
n.	45	46	47	48	49	50	51		-	-	-

**Table A2:** List of datasets for the damaged cases for selected $\alpha_{x}$.

Location	$\alpha_{x}\left(\%\right)$	Severity								
		1	2	3	4	5	6	7	8	9
d1	**1.0**	1	2	3	4	5	6	7	8	9
	**2.0**	10	11	12	13	14	15	16	17	18
	**3.0**	19	20	21	22	23	24	25	26	27
d2	**1.0**	28	29	30	31	32	33	34	35	36
	**2.0**	37	38	39	40	41	42	43	44	45
	**3.0**	46	47	48	49	50	51	52	53	54
d3	**1.0**	55	56	57	58	59	-	-	-	-
	**2.0**	60	61	62	63	64	-	-	-	-
	**3.0**	65	66	67	68	69	-	-	-	-
